# Measuring the effects of the new ECOWAS and WAEMU tobacco excise tax directives

**DOI:** 10.1136/tobaccocontrol-2020-055843

**Published:** 2020-09-28

**Authors:** Jean Tesche, Corne Van Walbeek

**Affiliations:** 1 Research Unit on the Economics of Excisable Products, School of Economics, University of Cape Town, Rondebosch, South Africa; 2 School of Economics, University of Cape Town, Rondebosch, South Africa

**Keywords:** taxation, economics, low/middle income country, public policy

## Abstract

**Background:**

In December 2017, the 15-member ECOWAS (Economic Community of West African States) and the 8-member WAEMU (West African Economic and Monetary Union, a subset of ECOWAS) passed new Tobacco Tax Directives. Both Directives increased the minimum ad valorem excise tax rate to 50%. In addition the ECOWAS Directive introduced a minimum specific tax (US$ 0.02/stick), but the WAEMU Directive did not. This paper examines the likely effects of these new Directives on cigarette prices, sales volumes and revenues.

**Method:**

Tax simulation models using comparable data were constructed for each of the 15 countries to estimate the effects of the ECOWAS and WAEMU Directives.

**Results:**

If the 15 ECOWAS members implement the ECOWAS Directive it would substantially increase the retail price of cigarettes (unweighted average 51%, range: 12% to 108%), decrease sales volumes (22%, range: −8% to −39%) and increase tax revenue (373%, range: 10% to 1243%). The impact of the WAEMU Directive on WAEMU countries’ cigarette prices (unweighted average +2%), sales volumes (−1%) and revenue (+17%) is likely to be minimal.

**Conclusions:**

The 2017 ECOWAS Directive, which adds a specific excise tax per pack, along with an increase in the ad valorem tax, substantially improves its members’ cigarette tax structure. The specific tax overcomes the weakness of the ad valorem excise tax, since it does not depend on import or ex-factory values, which comprise only a small part of the retail price in ECOWAS countries. We recommend that WAEMU countries adopt the ECOWAS Directive, rather than the WAEMU Directive.

## Introduction

Taxes are the single most effective measure to reduce tobacco use.^1^ Sub-Saharan Africa lags behind other WHO regions^2^ in tax levels even though 44 of its 47 countries are Parties to the Framework Convention on Tobacco Control (FCTC).^3^

**Figure 1:**
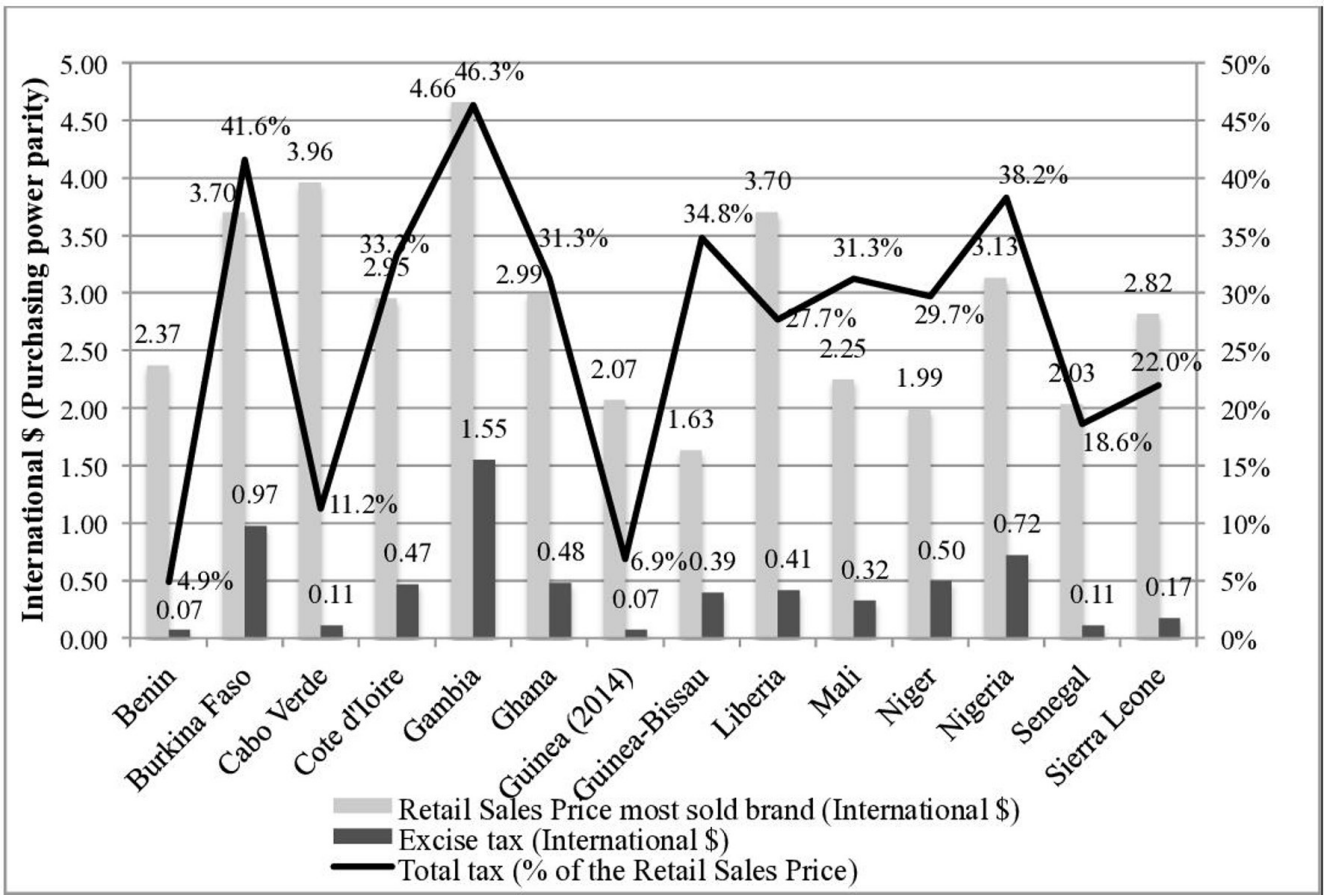
Retail price, excise tax and total tax share of the price of the most sold brand of cigarettes, 2018. Source: WHO, Global Tobacco Control Report, 2019.

ECOWAS member states have been even slower than the rest of the sub-Saharan African (AFRO) region to use tobacco taxes as an effective tobacco-control tool (figure 1). Of these, only Gambia has price and tax levels higher than the sub-Saharan African regional average.

While global smoking prevalence has fallen by more than 10 percentage points since 2000, the number of smokers in Africa is still growing, although from a relatively low base.^4^ The growth in the number of African smokers is mainly ascribed to the fact that the populations are young (the median age in most sub-Saharan African countries was under 14 years old in 2015)^5^ and growing,^6^ and that the tobacco industry is aggressively marketing their products in Africa.^7^ In addition, between 2008 and 2018, cigarettes became more affordable in 10 of the 12 ECOWAS countries for which data are available.^8^

The two economic blocs in Western Africa are the Economic Community of West African States (ECOWAS) and the West African Economic and Monetary Union (WAEMU). ECOWAS is a customs union, consisting of 15 countries (Benin, Burkina Faso, Cabo Verde, Cote d’Ivoire, the Gambia, Ghana, Guinea, Guinea-Bissau, Liberia, Mali, Niger, Nigeria, Senegal, Sierra Leone and Togo). In customs unions, goods traded within the union are not subject to import duties, and a common import duty is imposed on non-member country imports. WAEMU is both a customs and monetary union, consisting of eight of the ECOWAS countries (Benin, Burkina Faso, Cote d’Ivoire, Guinea-Bissau, Mali, Niger, Senegal and Togo). Member states share a common currency, and, therefore, monetary policy. Both ECOWAS and WAEMU adopted new tobacco tax directives in December 2017.^9 10^ Both consider harmonisation of tobacco excise taxes to be essential to the common markets. Harmonisation decreases incentives for cross-border shopping and illicit trade due to price difference between countries.

### ECOWAS Directive

The new ECOWAS Tobacco Tax Directive substantially increases the ad valorem excise tax rates and addresses systemic problems in the previous Directive.^11^ It increases the minimum ad valorem rate from 15% to 50% and adds a minimum specific (monetary amount) tax of US$ 0.02/stick (or US$ 0.40 per pack of 20 cigarettes). It also eliminates the maximum ad valorem tax rate of 100%. However, the tax base for the ad valorem excise tax did not change; it remains the import value for imported cigarettes and the ex-factory value for domestically produced cigarettes. The ex-factory value is the declared price when domestic products leave the factory. Import values are the declared price at customs, and include costs, insurance and freight (CIF). ECOWAS member states were given 3 years from 1 January 2018 to implement the new measures, so the new minimum taxes are to be in effect by 1 January 2021.

### WAEMU Directive

The new WAEMU Directive increases the minimum ad valorem tax rate from 15% to 50%. However, it retains a maximum ad valorem tax rate, which is increased from 45% to 150%.^10 12^ As with the ECOWAS Directive, the tax base continues to be the import or ex-factory value. Importantly, the WAEMU Directive does not add a specific tax amount. The WAEMU Directive was to be implemented by 31 December 2019.

Since WAEMU forms a subset of ECOWAS, the WAEMU member states are, in principle, subject to two substantially different directives. WAEMU countries have mostly complied with the WAEMU Directive. However, due to a lack of institutions and mechanisms to enforce compliance, some countries within ECOWAS and WAEMU have not complied with tax rates set in previous tobacco tax directives.^13 14^

This paper examines how the two directives will affect cigarette taxes, tax revenues, prices and the volume of sales (hereafter the ‘variables of interest’), should all member states implement them. The first set of simulations indicates the price and market changes for each of the 15 ECOWAS countries following tax changes made in accordance with the ECOWAS Directive. A second set considers the impact on the eight WAEMU member states if they were to implement the new WAEMU Directive minimum ad valorem rate of 50%. This is followed by a third set of simulations, should the WAEMU countries increase the ad valorem excise tax to the maximum rate of 150%.

## Methods

The simulation model was programmed in Excel and contains individual models for each of the 15 ECOWAS countries, which include differences in tax rates and structures, as well as different market segments. Each country model is similar to other tobacco simulation models, such as TaXSim^15^ and TETSiM.^16^ Each model consists of a base-year scenario reflecting the market and tax situation for each country at the outset, while the simulated scenarios indicate the impact of proposed tax changes.

The base model for each of the 15 countries incorporates tax rate and structure information for 2018, or the most recent available information. The first step decomposes the price per pack into its various tax and net-of-tax components. The base-year excise, value-added tax, customs duty and other tax rates (table 1) are applied to the appropriate tax bases. For all ECOWAS countries the tax base is the import value or ex-factory value per pack for domestic production. The net-of-tax price is the difference between the retail price and the sum of all taxes. It includes tobacco industry costs, retail and wholesale margins and profits. The per pack amounts are then multiplied by the total number of packs for total market value, excise and other tax revenues, and the net-of-tax amount. These calculations reproduce, or at least approximate, the base-year values.

**Table 1:** Tax rates for the base calculations

	Import duty (on imports from outside ECOWAS)	Specific excise per pack (local currency, US dollars)	Ad valorem excise rate	VAT rate	CIF or ex-factory value as percentage of retail price
Benin	20%		40%	18%	10.6%
Burkina Faso	20%		45%	18%	26.0% to 31.2%
Cabo Verde	37.9%		30%	15%	27.9% to 61.8%
Côte d'Ivoire	20%		33%	21%	30.3%
Gambia	20%	20 (US$0.43)		15%	13.4%
Ghana	20%		175%	17.5%	4.8% to 14.4%
Guinea	20%		20%	15%	17.9%
Guinea-Bissau	20%		45%	17%	24.2%
Liberia	5%		80%	10%	23.0%
Mali	20%		32%	18%	22.2%
Niger	20%		50%	19%	25.1%
Nigeria	60%	20 (US$0.06)	20%	5%	8.8% to 22.0%
Senegal	20%		65%	18%	20.2% to 23.1%
Sierra Leone	35%		35%	15%	9.3%
Togo	20%		50%	18%	12.4%

Ranges in the last column indicate the ex-factory or CIF shares for different price segments.

Sources: WHO GTCR 2019,^8^ Guinea: Word Bank,^25^ 2017, Cabo Verde-excise: country engagement 2020.

CIF, costs, insurance and freight; ECOWAS, Economic Community of West African States; GTCR, Global Tobacco Control Report; VAT, value-added tax; WAEMU, West African Economic and Monetary Union.

In the second step, taxes are adjusted to comply with the new Directives. For scenario 1, we apply the new minimum taxes from the ECOWAS Directive (50% ad valorem tax plus a specific tax of US$0.40 per pack) for the 15 ECOWAS countries. Scenarios 2A and 2B apply the new WAEMU minimum (50% ad valorem) and maximum (150% ad valorem) taxes on the eight WAEMU countries, respectively. Where the existing excise tax meets or exceeds the ad valorem minimums, as in Ghana, Liberia and Senegal, we assume that these countries will not change the ad valorem tax component, and will only introduce the specific component (for the ECOWAS Directive). The Gambia currently has a specific tax that is slightly higher than the new minimum; this also remains unchanged, and the new 50% minimum ad valorem is added. Based on these new taxes, and assuming that all other tax rates and the import, or ex-factory and net-of-tax values remain unchanged, the model calculates new retail prices for each of these scenarios. These new, higher prices decrease the quantity of sales.

The impact of the higher prices on cigarette sales is quantified by means of the price elasticity of demand. We use a price elasticity of demand of −0.7 for all countries (ie, a 1% increase in price results in a 0.7% decrease in cigarette sales). Globally, most estimates of the elasticity of demand for cigarettes are in the range of −0.4 to −0.8.^17–19^ We use the midpoint (or arc) formula to calculate the change in quantities consumed. This method is more appropriate when the retail price changes are large.^16^

Based on the simulated quantity consumed, the new taxes are used to calculate new excise and total tax revenue. The model calculates the new excise tax burden, by dividing the new excise tax amount by the new retail sales price.

Given the difficulties with data availability and consistency, we focus on the percentage changes in the variables of interest, rather than the absolute values. If, for example, sales volumes are substantially under-reported in the base scenario, the simulated sales and revenue numbers, in absolute terms, would be biassed downwards, but the percentage change in these variables is not affected, as the under-reporting applies to both the base scenario and the simulated scenario.

### Data

The main sources of data for the models used in this analysis are the WHO Report on the Global Tobacco Epidemic, 2019 (hereafter Global Tobacco Control Report or GTCR),^8^ United Nations Comtrade database,^20^ and GlobalData.^21^ The GTCR and Comtrade are used for tobacco tax and trade data, respectively, because the data are comparable across countries and over time. We used the most recent data sources available, usually from 2017 or 2018. Earlier data are used for Guinea, Guinea-Bissau and Liberia owing to the lack of reporting. Table 2 shows the data sources for each country. Given the multiple sources, the data for a country may not be from the same year.

**Table 2:** Data sources and base year

	Import quantity and price: Comtrade	Total domestic supply	Price of the most sold brand: GTCR
Benin	2017	Imports only	2017
Burkina Faso	2017	GTCR (excise revenue/ (excise revenue/pack)	2017
Cabo Verde	2018	SCT annual report^23^	2017
Cote d'Ivoire	2017	GlobalData 2017	2017
Gambia	2017	Imports only	2017
Ghana	2018	Imports only	2018
Guinea	2015	Imports only	2014
Guinea-Bissau	2016	Imports only	2016
Liberia	2016	Imports only	2016
Mali	2017	Imports only	2018*
Niger	2017	Imports only	2018*
Nigeria	2018	GlobalData, 2018	Euromonitor^26^
Senegal	2017	GlobalData, 2017	2018*
Sierra Leone	2017	Imports only	2018*
Togo	2017	Imports only	2018*

Shaded countries are also members of WAEMU.

*Same price reported in 2016 and 2018, GTCR, 2019^8^.

GTCR, Global Tobacco Control Report; SCT, Sociedade Caboverdiana de Tabacos; WAEMU, West African Economic and Monetary Union.

### Prices

Retail prices of the most-sold brand (mostly for 2018) are from the 2019 GTCR. These prices are used as a proxy for the average retail sales price for the nine countries with data for the entire market only as well as for the mid-level segment for the other six countries. GTCR prices for premium and economy brands are used for those market segments, respectively. For Burkina Faso, Cabo Verde, Nigeria and Senegal, imported cigarettes are premium brands. The retail price of the most-sold brand is used for both imports and domestic production in Cote d’Ivoire, since nearly all cigarettes were sold at the same price. Nigeria uses GTCR premium prices for both imported and domestic premium cigarettes.

### Import quantities

Comtrade data were used for import quantities for all countries. Data are available for most countries for 2017, and for 2018 for Cabo Verde, Ghana and Nigeria (table 2). The most recent Comtrade data for Guinea are from 2015. No import data were reported for Guinea-Bissau and Liberia, so the sum of all cigarette exports to each of those countries for the most recent year available, 2016, was used as a proxy. The number of cigarette packs imported is calculated using the net weight of total imports, in grams, divided by 20 sticks per pack, assuming that each cigarette weighs 1 g. A recent OECD (Organisation for Economic Co-operation and Development) survey of member countries notes that a cigarette typically weighs around 1 g per stick, even with varying amounts of tobacco.^22^

### Import prices

In all cases, except Cabo Verde, the import value (CIF) is derived from Comtrade data (see table 1 for the percentage of CIF or ex-factory values in the retail price). The total value of cigarette imports, in US dollars, is divided by the number of packs imported. To convert to local currencies, we use the Comtrade conversion factor (the US$/local currency exchange rate) for the relevant year. For Cabo Verde, we use import and ex-factory values published in the Annual Report of the main producer and importer of cigarettes, Sociedade Caboverdiana de Tabacos (SCT).^23^

### Import taxes and levies

Imports from countries within ECOWAS (and therefore WAEMU) are free from duties. We use the sum of reported imports from non-ECOWAS countries from Comtrade to calculate the proportion of cigarette imports that are subject to customs duties. Although customs unions are meant to impose uniform external tariffs, in practice those in ECOWAS countries differ^14^ (table 1). All WAEMU countries follow the common WAEMU tariff of 20%, with the exception of Senegal, which adds a 20% surcharge. Both customs unions impose additional fees on non-ECOWAS imports.

### Domestic production data

Cigarettes are not produced in 10 of the 15 countries, so imports constitute total supply. For the countries that both import and produce cigarettes (Burkina Faso, Cabo Verde, Cote d’Ivoire, Nigeria and Senegal, table 2), production for domestic use is the difference between total domestic supply and imports. Production data for Cabo Verde comes from the SCT 2018 Annual Report.^23^ Total domestic supply for Cote d’Ivoire, Nigeria and Senegal is taken from GlobalData. For Burkina Faso, total domestic supply is estimated by total tobacco tax revenue, GTCR 2019^8^ divided by the product of the excise tax rate and the calculated CIF price per pack. For Mali, no recent data on domestic production was available, so all cigarettes are assumed to be imported. This almost certainly understates total supply in Mali since there has recently been new investment in domestic production.^24^

### Market shares

Because of lack of data on the size of market segments, 9 of the 10 countries that consume only imported cigarettes are considered to have only one market segment. The exception is Ghana, which has three. For Burkina Faso, Cote d’Ivoire and Senegal, imports are considered as a separate market category from domestically produced cigarettes. For Cabo Verde, imports make up one of the three market segments. For Nigeria, there are three market segments, but premium brands are further divided into import and domestic segments.

## Results

The impact of the new ECOWAS Directive is substantial (table 3). Retail prices are expected to increase by an average (all are simple, unweighted averages) of 51% (from 12% in the Gambia to more than 100% in Guinea). Cigarette sales are expected to decrease by an average of 22% (from −8% in the Gambia to −39% in Guinea). Excise tax revenue is predicted to increase by an average of 373% (from 10% in the Gambia to more than 1000% in Guinea and Sierra Leone). The tax burden is expected to increase from an average of 13% in the base year (4% in Benin, Guinea and Sierra Leone to 33% in the Gambia) to 37% (from 31% in Togo to 46% in Guinea and Nigeria).

**Table 3:** Simulation 1 results: cigarette excise taxes increased to ECOWAS minimums

	Retail sales price (RSP)	Sales volumes	Excise revenue	Total tax revenue	Excise % of RSP before tax increase	Excise % of RSP after tax increase
Benin	56%	−27%	774%	532%	4%	33%
Burkina Faso	38%	−11%	195%	142%	14%	33%
Cabo Verde	35%	−19%	166%	110%	14%	32%
Cote d'Ivoire	48%	−24%	177%	125%	15%	37%
Gambia	12%	−8%	20%	16%	33%	39%
Ghana	42%	−22%	133%	74%	17%	36%
Guinea	108%	−39%	1243%	474%	4%	46%
Guinea-Bissau	57%	−27%	248%	142%	13%	39%
Liberia	35%	−14%	130%	112%	19%	37%
Mali	40%	−10%	346%	176%	8%	30%
Niger	56%	−27%	239%	165%	13%	39%
Nigeria	73%	−32%	152%	127%	22%	46%
Senegal	53%	−26%	129%	68%	15%	31%
Sierra Leone	70%	−15%	1192%	609%	4%	38%
Togo	46%	−23%	446%	325%	6%	31%
**Unweighted average**	**51%**	−**22%**	**373%**	**213%**	**14%**	**37%**

ECOWAS, Economic Community of West African States.

Table 4 indicates the likely impact on the variables of interest should the WAEMU countries increase their excise taxes to the minimum of 50%, as stipulated by the new WAEMU Directive (Simulation 2A). The new Directive would have no impact on Niger, Senegal and Togo, as these countries’ initial excise tax rates were already at or above 50%. The other five countries would be required to raise their tax rates. The retail price is expected to increase, on average, by 2%, while sales volumes are expected to decrease, on average, by 1%. The average increase in excise tax revenue of 17% is small compared with the average increase of 373% if the ECOWAS Directive is implemented. The average excise tax burden increases from 11% to 13%.

**Table 4:** Simulation 2A results: excise taxes increased to WAEMU minimum (50%)

	Retail sales price (RSP)	Sales volumes	Excise revenue	Excise % RSP before tax increase	Excise % RSP after tax increase
Benin	1%	−1%	24%	4%	5%
Burkina Faso	2%	−1%	10%	14%	15%
Cote d'Ivoire	8%	−5%	36%	15%	20%
Guinea-Bissau	2%	−1%	10%	13%	14%
Mali	6%	−2%	53%	8%	13%
Niger	0%	0%	0%	13%	15%
Senegal	0%	0%	0%	15%	15%
Togo	0%	0%	0%	6%	6%
**WAEMU**	**2%**	−**1%**	**17%**	**11%**	**13%**

WAEMU, West African Economic and Monetary Union.

If all WAEMU countries increased their excise taxes to the new maximum rate of 150% (Simulation 2B), there would be a substantially greater impact on all variables of interest (table 5). Prices are expected to increase by an average of 33% (from 14% in Benin to 60% in Cote d’Ivoire), sales to decrease by an average of 15% (from −9% in Benin and Togo to −28% in Cote d’Ivoire) and excise tax revenues to increase by an average of 194% (from 89% in Senegal to more than 320% in Mali). The average excise tax burden is expected to increase from 11% to 28%. Even at the maximum allowed ad valorem rate of 150% the average excise tax burden does not reach the 37% average excise tax burden achieved had the ECOWAS Directive been implemented.

**Table 5:** Simulation 2B results: excise taxes increased to WAEMU maximum (150%)

	Retail sales price (RSP)	Sales volumes	Excise revenue	Excise % RSP before tax increase	Excise % RSP after tax increase
Benin	14%	−9%	242%	4%	14%
Burkina Faso	38%	−11%	197%	14%	33%
Cote d'Ivoire	60%	−28%	210%	15%	40%
Guinea-Bissau	35%	−19%	170%	13%	32%
Mali	37%	−10%	323%	8%	29%
Niger	31%	−17%	148%	13%	33%
Senegal	33%	−18%	89%	15%	27%
Togo	15%	−9%	172%	6%	17%
**WAEMU**	**33%**	−**15%**	**194%**	**11%**	**28%**

WAEMU, West African Economic and Monetary Union.

### Sensitivity analysis

The assumption that only the tax increase changes retail prices implies that cigarette producers do not change net-of-tax prices to influence demand and/or to protect their profits after a tax increase. In fact, the tobacco industry can change any of the non-tax components, which affects prices and sales volumes. In table 6 we present summarised results of changes in the industry margin in response the new ECOWAS and WAEMU Tax Directives. A decrease in industry margins implies that the tobacco industry bears a part of the additional tax amount, reducing the increase in the retail price and decreasing the reduction in sales volumes compared with full tax pass-through. An increase in industry margins has the opposite effect; retail prices increase by more than the increase in the tax, so sales volumes decrease and tax revenues increase by less than with full pass through. For countries implementing the ECOWAS Directive, industry pricing decisions do not change the overall qualitative results of the model. However, because the impact of the WAEMU Directive on the variables of interest is so small, industry pricing decisions can have a relatively larger impact, and even overturn some of the (modest) gains achieved by the Directive.

**Table 6:** Sensitivity analysis: percentage of pass through–averages

Pass through (%)	Retail sales price	Sales volumes	Excise revenue	Total tax revenue
ECOWAS (15) minimum rates (50%+specific=US$0.40)				
−10%	45%	−20%	383%	219%
Full pass through	51%	−22%	373%	213%
10%	57%	−23%	364%	207%
WAEMU (8) minimum rate (50%)				
−10%	−3%	2%	10%	14%
Full pass through	2%	−1%	17%	10%
10%	8%	−4%	3%	7%

ECOWAS, Economic Community of West African States; WAEMU, West African Economic and Monetary Union.

We also performed sensitivity analysis using a higher (−0.9) and a lower (−0.5) price elasticity of demand than the model’s value of −0.7 (table 7 and the online supplemental file for individual country results). The price elasticity of demand is a crucial parameter in determining the impact of tax and price changes on sales volumes. Sales volumes will decrease by 26% if the price elasticity is −0.9 and by 16% if the elasticity is −0.5, compared with 22% with the model’s price elasticity of −0.7. The price elasticity estimates of −0.9 and −0.5 are expected to increase average excise revenue by 337% and 412%, respectively, compared with the model scenario of 373%. Given the large increases in taxes required by the ECOWAS Directive for most countries, the choice of elasticity does not alter the direction and the approximate magnitude of the changes.

10.1136/tobaccocontrol-2020-055843.supp1 Supplementary data

**Table 7:** Sensitivity analysis: price elasticity–averages

Elasticity	Retail sales price	Sales volumes	Excise revenue	Total tax revenue
ECOWAS (15) minimum rates (50%+specific=US$0.40)				
−0.9	51%	−26%	337%	191%
−**0.7**	**51%**	−**22%**	**373%**	**213%**
−0.5	51%	−16%	412%	238%
WAEMU (8) minimum rate (50%)				
−0.9	2%	−2%	16%	10%
−**0.7**	**2%**	−**1%**	**17%**	**10%**
−0.5	2%	−1%	17%	11%

ECOWAS, Economic Community of West African States; WAEMU, West African Economic and Monetary Union.

Changing elasticities when taxes are set at the WAEMU minimum makes little difference to the results because the expected price changes are so small.

## Discussion

**Figure 2:**
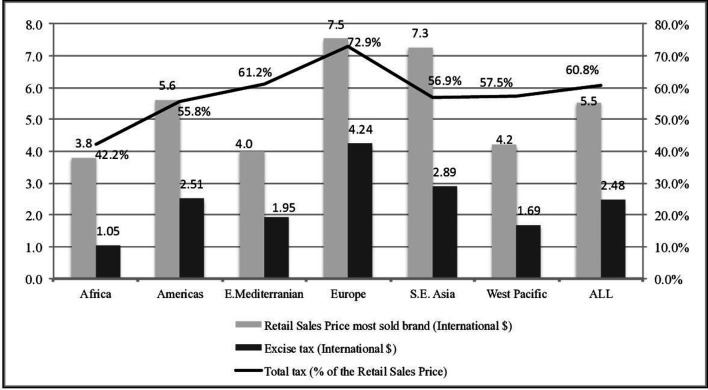
Retail sales price, excise tax and total tax share of the most sold brand of cigarettes, by WHO Regional Group, 2018 Source: WHO GTCR, 2019. GTCR, Global Tobacco Control Report.

The introduction of a specific tax (US$0.40 per pack) in the ECOWAS Directive has a much greater impact on prices and sales volumes than the increase in the ad valorem rate to 50% (or even to 150%) required by the WAEMU Directive. Implementation of the ECOWAS Directive increases the average unweighted excise tax burden from an average of 13% to 37%. This would be a major change and would make the excise tax burden in the ECOWAS countries higher than the 28% average in the WHO African region in 2018. However, tax shares would still be lower than the global average of 45% (figure 2).

Increasing taxes for the eight WAEMU countries to a minimum of 50% would have a very small effect (Simulation 2A). In three of the eight WAEMU countries, the ad valorem rate at the outset was 50% or more; for those countries the new WAEMU minimum will simply maintain the status quo.

Increasing the ad valorem tax component to WAEMU maximum of 150% of import or ex-factory values clearly shows the limitations of increasing the tax rate in a purely ad valorem tax system. It would bring the unweighted average excise tax burden to the 2018 average of 28% for sub-Saharan Africa, compared with 37% should the ECOWAS Directive be adopted.

The declared import or ex-factory values in the ECOWAS countries are very low, with a median share of the retail price of about 22% (table 1). Even substantial increases in the ad valorem rate (Simulation 2B) will have only a modest impact. As long as the tax base is the import or ex-factory value, ad valorem rate increases will have little impact. The tobacco industry has an incentive to declare even lower import and/or ex-factory values when faced with a substantially higher ad valorem tax rate.

### Limitations

The biggest limitation is the lack of reliable country data. The use of Comtrade and WHO GTCR data ensures a level of consistency, but relies on reporting from countries. The validity of the data depends on a country’s data management.

Another limitation is that one of the basic assumptions of the model is that there is no change in existing illicit trade levels. The increases in revenues and decreases in sales volumes may be overestimated if smokers switch to cheaper cigarettes. If consumption shifts to illegal cigarettes or other tobacco products, both revenues and legal sales would decrease.

## Conclusions

Implementation of the 2017 ECOWAS Directive in 2021 would improve the tax structure of these countries by adding a specific excise tax along with the increase in the ad valorem tax. Because the US$0.40 per pack specific tax does not depend on import or ex-factory values, it will overcome the major weakness of the ad valorem tax in the previous Directive.

Many countries, in Africa and elsewhere, use the import or ex-factory price as the ad valorem tax base, probably due to constraints on valuing and collecting excise taxes at the retail level because of large informal sectors. However, it is difficult to increase ad valorem rates sufficiently to have a substantial impact on prices, and therefore on sales volumes, from such a low base. A specific tax has the additional advantage of being easier to collect, since it depends on quantities rather than values. It is also be less susceptible to manipulation as it does not depend on the valuation of imports or production.

We recommend that ECOWAS member states implement the new Directive in January 2021 in order to improve health, to pre-empt increases in smoking prevalence resulting from aggressive marketing to their young, growing populations, and to increase revenues. WAEMU member countries should opt to comply with the ECOWAS rather than the WAEMU Directive.

**What this paper adds**

- Tobacco taxes are lower in Economic Community of West African States (ECOWAS) (including the West African Economic and Monetary Union (WAEMU)) countries than the global average as well as the average for sub-Saharan Africa.
- In December 2017 both ECOWAS and WAEMU adopted new Directives on tobacco taxation. What has been missing is an analysis of the impact on price, sales volumes (with its implications for public health) and revenues of the two Directives on each of these two blocs’ member countries.
- This paper constructs cigarette tax models for the 15 ECOWAS countries using each country’s tax rates and structures. We analyse the effects of implementing the two Directives on prices, sales volumes and tax revenues. The paper highlights the weakness of a purely ad valorem system that uses the import or ex-factory values as a tax base, and the advantage of a specific tax that is independent of the value of the product.

## Data Availability

Data are available in a public, open access repository. Data are available upon reasonable request. WHO Report on the Global Tobacco Epidemic, 2019: Monitoring tobacco use and prevention policies. Geneva, Switzerland: WHO, 2019. https://www.who.int/tobacco/global_report/en/. United Nations Comtrade. United Nations Commodity Trade Statistics Database. https://comtrade.un.orgplc. GlobalData, https://www.globaldata.com Sociedade Caboverdiana de Tabacos, S.A. Annual Report and Accounts for 2018, http://www.bcv.cv/vPT/Mercado_de_Capitais/Sistema/Emitentes/Prestação_de_Contas/Documents/Relatórios_e_Contas_em_2019/Relatório__Contas_2018_SCT_FINALcompactado.pdf
