# Supplementary material for: Measuring the effects of the new ECOWAS and WAEMU tobacco excise tax directives
Source: Tob Control. 2020 Sep 28;30(6):668–74. doi: 10.1136/tobaccocontrol-2020-055843 (PMC8543226; doi:10.1136/tobaccocontrol-2020-055843)
Supplement: Supplementary data [file tobaccocontrol-2020-055843supp001.pdf]

**Measuring the Effects of the New ECOWAS and WAEMU Tobacco Excise Tax Directives**

**Supplementary File**

**Table S-1: Simulation 1: cigarette excise taxes increased to ECOWAS minimums Specific tax $0.40 per pack + 50% minimum ad valorem--Price elasticity = -0.9**

| | Retail Sales Price (RSP) | Consumption | Excise Revenue | Total Tax Revenue | Excise % RSP before tax increase | Excise % RSP after tax increase |
|---|---|---|---|---|---|---|
| Benin | 56% | -33% | 699% | 478% | 4% | 33% |
| Burkina Faso | 37% | -14% | 186% | 135% | 14% | 33% |
| Cabo Verde | 35% | -24% | 151% | 98% | 14% | 32% |
| Cote d'Ivoire | 48% | -30% | 156% | 107% | 15% | 37% |
| Gambia | 12% | -10% | 17% | 13% | 33% | 39% |
| Ghana | 43% | -28% | 117% | 62% | 17% | 36% |
| Guinea | 108% | -48% | 1053% | 393% | 4% | 46% |
| Guinea-Bissau | 57% | -33% | 217% | 120% | 13% | 39% |
| Liberia | 35% | -14% | 130% | 112% | 19% | 37% |
| Mali | 40% | -13% | 333% | 168% | 8% | 30% |
| Niger | 56% | -33% | 210% | 142% | 13% | 39% |
| Nigeria | 73% | -39% | 125% | 103% | 22% | 46% |
| Senegal | 53% | -32% | 110% | 54% | 15% | 31% |
| Sierra Leone | 70% | -19% | 1137% | 579% | 4% | 38% |
| Togo | 46% | -29% | 406% | 294% | 6% | 31% |
| **ECOWAS** | **51%** | **-26%** | **337%** | **191%** | **14%** | **37%** |

**Table S-2: Simulation 1: cigarette excise taxes increased to ECOWAS minimums**
**Specific tax $0.40 per pack + 50% minimum ad valorem--Price elasticity = -0.5**

| | Retail Sales Price (RSP) | Consumption | Excise Revenue | Total Tax Revenue | Excise % RSP before tax increase | Excise % RSP after tax increase |
|---|---|---|---|---|---|---|
| Benin | 56% | -20% | 855% | 591% | 4% | 33% |
| Burkina Faso | 38% | -8% | 205% | 150% | 14% | 33% |
| Cabo Verde | 34% | -14% | 183% | 123% | 14% | 32% |
| Cote d'Ivoire | 48% | -18% | 200% | 143% | 15% | 37% |
| Gambia | 12% | -6% | 23% | 19% | 33% | 39% |
| Ghana | 42% | -17% | 150% | 86% | 17% | 36% |
| Guinea | 108% | -30% | 1456% | 565% | 4% | 46% |
| Guinea-Bissau | 57% | -20% | 281% | 164% | 13% | 39% |
| Liberia | 35% | -14% | 130% | 112% | 19% | 37% |
| Mali | 40% | -8% | 360% | 185% | 8% | 30% |
| Niger | 56% | -20% | 271% | 190% | 13% | 39% |
| Nigeria | 72% | -24% | 181% | 153% | 22% | 46% |
| Senegal | 53% | -19% | 150% | 83% | 15% | 31% |
| Sierra Leone | 70% | -11% | 1251% | 642% | 4% | 38% |
| Togo | 46% | -17% | 489% | 358% | 6% | 31% |
| **ECOWAS** | **51%** | **-16%** | **412%** | **238%** | **14%** | **37%** |
